# Expanding the prion concept to cancer biology: dominant-negative effect of aggregates of mutant p53 tumour suppressor

**DOI:** 10.1042/BSR20130065

**Published:** 2013-07-25

**Authors:** Jerson L. Silva, Luciana P. Rangel, Danielly C. F. Costa, Yraima Cordeiro, Claudia V. De Moura Gallo

**Affiliations:** *Instituto de Bioquímica Médica, Rio de Janeiro, RJ 21941-902, Brazil; †Instituto Nacional de Ciência e Tecnologia de Biologia Estrutural e Bioimagem, Rio de Janeiro, RJ 21941-902, Brazil; ‡Faculdade de Farmácia, Universidade Federal do Rio de Janeiro, Rio de Janeiro, RJ 21941-902, Brazil; §Departamento de Genética, IBRAG, Universidade do Estado do Rio de Janeiro, Rio de Janeiro, Brazil

**Keywords:** amyloid, cancer, p53, prions, protein aggregation, protein misfolding, AD, Alzheimer’s disease, AFM, atomic force microscopy, ALS, amyotrophic lateral sclerosis, APR, aggregation-prone region, CTD, C-terminal domain, DBD, DNA-binding domain, HSP, heat-shock protein, Mdm2, murine double minute 2, MTT, 3-(4,5-dimethylthiazol-2-yl)-2,5-diphenyl-2*H*-tetrazolium bromide, PMD, protein misfolding disease, WT, wild-type

## Abstract

p53 is a key protein that participates in cell-cycle control, and its malfunction can lead to cancer. This tumour suppressor protein has three main domains; the N-terminal transactivation domain, the CTD (C-terminal domain) and the core domain (p53C) that constitutes the sequence-specific DBD (DNA-binding region). Most p53 mutations related to cancer development are found in the DBD. Aggregation of p53 into amyloid oligomers and fibrils has been shown. Moreover, amyloid aggregates of both the mutant and WT (wild-type) forms of p53 were detected in tumour tissues. We propose that if p53 aggregation occurred, it would be a crucial aspect of cancer development, as p53 would lose its WT functions in an aggregated state. Mutant p53 can also exert a dominant-negative regulatory effect on WT p53. Herein, we discuss the dominant-negative effect in light of p53 aggregation and the fact that amyloid-like mutant p53 can convert WT p53 into more aggregated species, leading into gain of function in addition to the loss of tumour suppressor function. In summary, the results obtained in the last decade indicate that cancer may have characteristics in common with amyloidogenic and prion diseases.

## INTRODUCTION

p53 is a tetrameric nuclear phosphoprotein that plays an essential role in the prevention of cancer development by inducing cell-cycle arrest or apoptosis in response to a variety of stress signals, such as DNA damage. Disruption of the p53 network usually has severe consequences that favour cell survival and tumour progression [1–3]. p53 is regulated primarily by the ubiquitin ligase Mdm2 (murine double minute 2), which binds p53 and targets it for degradation in proteasomes [4].

**Figure 1:**
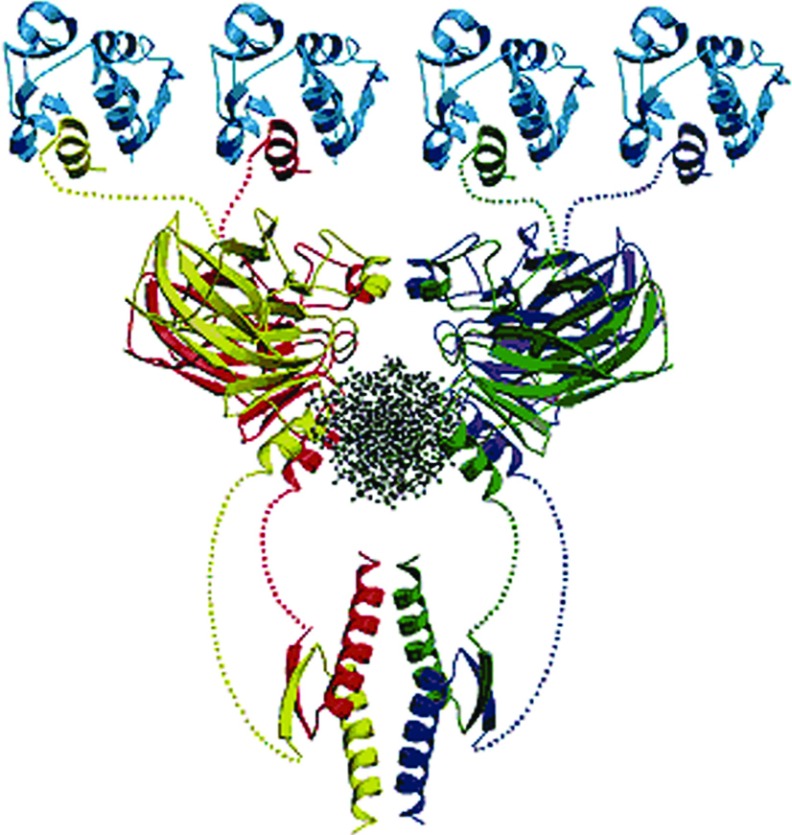
Structure of tetrameric p53 (each monomer coloured in red, yellow, blue or green) bound to a DNA sequence (black, middle) and to four Mdm2 molecules (top, blue) Figure adapted from Pennisi E. Filling in the blanks in the p53 protein structure (1996) Science 274 (5289) 921–922. Reprinted with permission from AAAS.

The human p53 protein comprises 393 amino acid residues and three main functional regions: the N-terminal activation domain, which is able to interact with a variety of proteins; the CTD, responsible for tetramerization; and the core domain (p53C) comprising residues 94–312 that constitute the sequence-specific DBD (DNA-binding region) of the protein [5,6] (Figure 1). More than 90% of the point mutations in p53 that are related to malignancy are found in this segment [7].

Mutations in the p53 gene (*TP53*) are frequently associated with increased susceptibility to cancer development. Inactivation of p53-regulated pathways has been described in over 50% of all human cancers, making them interesting targets for cancer therapies [1,7–10]. Most of these mutations result in the expression of a p53 protein that has lost its WT (wild-type) functions, gained additional functions and may exert a dominant-negative regulation over the WT p53 [11]. Mutant p53 can acquire oncogenic functions, independent of WT p53, to drive mechanisms of cell migration, invasion and metastasis [11,12]; these events will be addressed below.

In addition to the loss of protein function caused by somatic mutations, aggregation of p53 in cells can also inactivate the WT protein, leading to malignancy [10]. Several cancer cells exhibit abnormal accumulation of WT or mutant p53 either in the cytoplasm or in the nucleus. This accumulation has already been described in neuroblastoma, retinoblastoma, breast and colon cancer cells [13–15]. There is evidence that the three functional domains of p53 could form amyloid-like aggregates [10,16–20], which leads us to speculate that p53 amyloid formation might participate in the malignant process [10,16].

The p53 protein has been extensively studied at both structural and functional levels. In this review, we outline how ordered and amyloid-like aggregates of the mutant p53 tumour suppressor might play a role in cancer development. We also discuss the pathways through which misfolded p53 could divert native proteins into aggregates, and how the mutant form, with its greater propensity for aggregation, would lead to a dominant-negative effect. These findings may reveal the biological significance of the prion-like behaviour of oncogenic p53 mutants and help to develop new strategies to disrupt the formation of aggregates. This approach would be particularly attractive for possible therapeutic applications in cancer and other PMDs (protein misfolding diseases).

### *TP53* mutations and mutant p53s

Mutations in *TP53* are very frequent in cancer and it is now clear that their occurrence is associated with bad prognosis, especially in breast cancer [21,22]. As a master regulator, p53 is at the centre of a node of complex pathways involved in cellular stress responses, including the maintenance of genomic integrity; based on these functions, Lane coined p53 as the guardian of the genome [23]. Inactivation of p53 leads to genomic instability and other hallmark features of cancer cells, such as resistance to apoptosis [24,25]. However, this is not the full story. In addition to the suppression of p53 activity, the presence of mutations in *TP53* may change the protein functionality to a dominant-negative form, sequestering WT p53 and other proteins or acquiring new oncogenic roles [26]. When p53 was discovered in 1979, it was described as an oncogenic protein associated with SV40 (simian virus 40)-transformed cells. Later, it was demonstrated that a mutant form, not WT p53, acted as a tumour suppressor. The discrepancies of the initial discoveries showing oncogenic or anti-oncogenic activities of p53 were later linked to the presence of either mutant or WT p53 forms in the different models. The results obtained in the past reinforce the observation that some mutant p53 proteins accumulate and have oncogenic roles during carcinogenesis [26,27]. For example, in 1979, De Leo and collaborators detected expression of a new cancer-related antigen, murine p53, in chemically induced sarcomas in mice; this protein was apparently absent in normal non-transformed cells, such as adult mouse fibroblasts, lymphoid cells, or haematopoietic cells [28]. A few years later, it was reported that the p53 ‘oncogene’ had the effect of immortalizing cells, which was increased by mutational events [29]. A common feature of these first reports was the observation of p53 accumulation in cells and its interaction with viral proteins [26,27].

**Figure 2:**
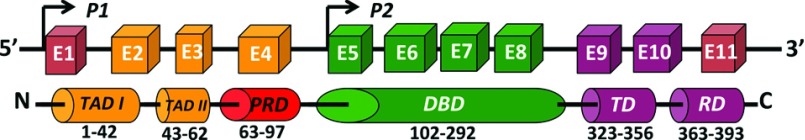
The human *TP53* gene based on IARC TP53 Mutation Database (www-p53.iarc.fr/) E, exon; P, promoter; TAD, transactivation domain; PRD, proline-rich domain; DBD, DNA-binding domain; TD, oligomerization domain; RD, regulation domain. The numbers in the bottom indicate amino acid residues that compose each p53 domain.

The human *TP53* gene [OMIM (Online Mendelian Inheritance in Man) 191170] is located at 17p13.1 and is composed of 11 exons (Figure 2). It has two transcription start sites in exon 1 and alternative splicing sites in intron 2 and between exons 9 and 10. Moreover, an internal promoter and transcription start site was described in intron 4 [30,31]. A common ancestral gene is believed to have existed in primitive organisms and then duplicated during evolution resulting in a family of genes composed of *TP53*, *TP63* and *TP73*. p53-related proteins are present in species from worms to humans. In contrast with other tumour suppressors, *TP53* mutations are characteristically point mutations instead of complete or partial deletions, such as observed in *Rb* and *BRCA1* [32]. These mutations are very diverse in nature, and, in human cancers, they are predominantly located in exons 5–8, which correspond to the p53 DBD (Figure 2). In fact, a number of these mutations are informative about the mutagenic agent involved in the process of carcinogenesis. Such association is clearly observed with the p.R249S mutation in hepatocellular carcinomas and aflatoxin-B1 exposure [33,34]. The highly detected presence of G>T transversions in lung cancer of tobacco smokers is also remarkable [35]. Evaluation of *TP53* mutations as biomarkers of mutagenic events is an advantageous scientific utilization of *TP53* mutational spectrum data. A comprehensive list of studies and databases is provided by the IARC *TP53* database and other databases [see IARC TP53 Mutation Database: www-p53.iarc.fr/; Database of Germline p53 Mutations: www.lf2.cuni.cz/projects/germline_mut_p53.htm; TP53 Mutation Database: http://p53.fr/; and p53 Mutations and Cancer: http://p53.free.fr/].

Interestingly, only *TP53* mutations that induce significant structural and functional changes in p53 will generate cell proliferative advantage and genomic instability, resulting in biological selection [26]. Such biological selection is intrinsically associated to the natural history of tumours, and the presence of mutant p53 is frequently linked to poor patient prognosis. Recently, Shah et al. showed that *TP53* is the most frequently mutated gene in tumours of triple negative (ER−, PR− and HER2−) breast cancer [36], considered an aggressive class of breast cancer. In a study dedicated to correlating the loss-of-function mutations in *TP53* with breast cancer prognosis, the authors found that mutations are more frequent in high-grade, large-size, node-positive cases and also in oestrogen- and progesterone-receptor-negative tumours [22]. Additionally, the different mutations were not equal in relation to their associated clinical characteristics; for example, p.R248W was associated with poorer prognoses [22]. Therefore at least in breast cancer, mounting evidence suggests that *TP53* mutations act by driving oncogenic aggressiveness and different mutations are not equivalent in this process [37].

In human cancers, the most common p53 mutants are derived from missense somatic mutations at ‘hot-spot’ residues located at the DBD. Some of the most frequent mutations include R175H, a structural mutant, and R248Q, R273H, R248W, R273C and R282W, which are contact mutants that have misfunctional interactions with the target DNA [32]. It is currently known that mutant p53s are characteristically stable and accumulate in cancer cells. This accumulation, however, is not direct proof of the presence of mutant p53 because WT p53 may also accumulate. Additionally, null p53 mutants do not accumulate, so the absence of accumulation is not direct proof of the lack of p53 mutations [38]. Altered protein stability and accumulation is of key importance to the aggregation state and, consequently, to the dominant-negative effect [10,19,20].

In general, p53 mutants may be classified according to their functional alterations: neutral, negative, dominant-negative or gain-of-function [32]. The last two classes are of great interest and recent studies show that complex interactions between the p53 variants and other proteins, such as transcription factors and the p53 paralogues p63 and p73, might participate in cancer genesis and evolution [11].

Gain-of-function of mutant p53 in cancer cells was well established by Di Como et al. in 1999 [39]. The authors demonstrated that both p.R175H and p.R248W decrease the ability of p73 to promote apoptosis. More recently, Muller et al. 2009 showed that overexpressed mutant p53 promotes invasion and metastasis through inhibition of p63 [40]. Evidence on the role of p63 in modulating EGFR (epidermal growth factor receptor)/integrin signalling and the association between mutant p53 and increased cell migration both *in vivo* and *in vitro* were also previously presented [40].

An interesting example of the connection between mutant p53s and cancer development is their role in the control of miRNA (microRNA) expression [41]. Ectopic expression of mutant p53s (R273H, R175H and C135Y) through stable transfection into the p53 knocked-down HEC-50 cell line, a cell line derived from endometrial cancer, showed that these mutants repressed the expression of miR-130b and triggered ZEB1-dependent epithelial–mesenchymal transition and cancer cell invasion [42]. Thus, mutant p53 may interact with diverse gene promoters in addition to its known interactions with different proteins. Such activities illustrate the flexibility and multifaceted functions of p53 variants.

### PMDs and the prion concept

**Figure 3:**
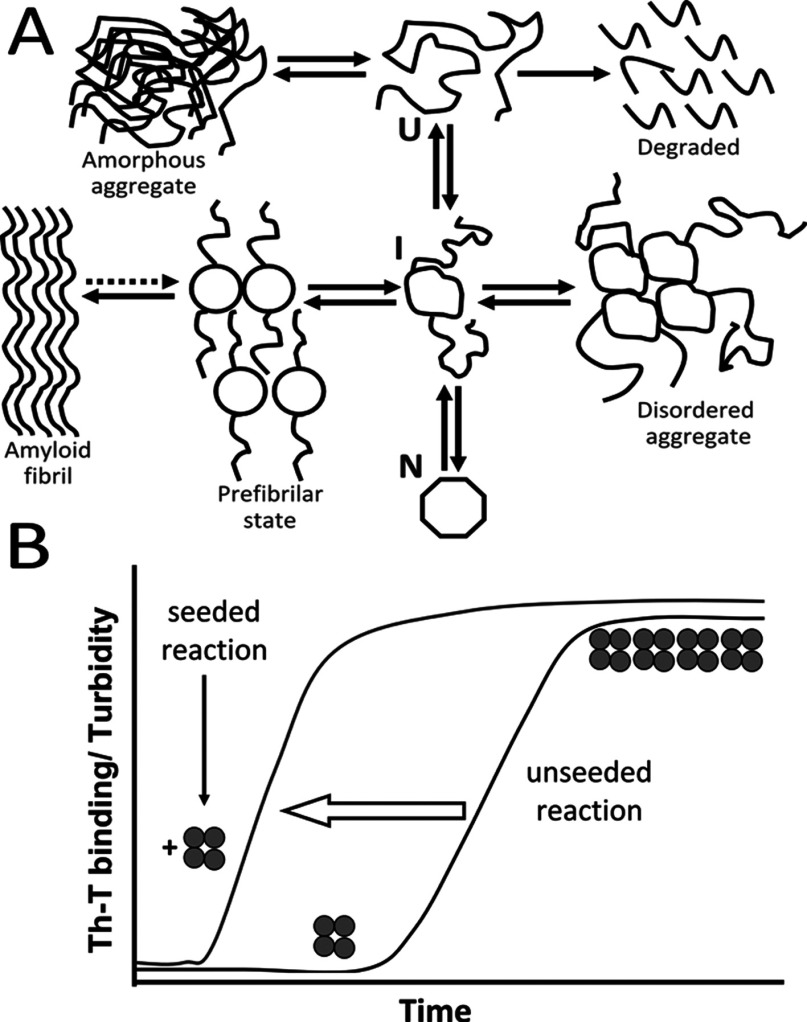
(A) Productive and unproductive protein folding pathways U, unfolded protein; I, intermediate folding state; N, native protein. Reprinted from Current Opinion in Structural Biology, 9(1), Christopher M Dobson, Martin Karplus, The fundamentals of protein folding: bringing together theory and experiment, 92–101, Copyright (1999), with permission from Elsevier. (**B**) **Seeding-nucleation aggregation process**. The unseeded reaction proceeds with a lag phase during which a seed is formed. After the formation of the initial seed (oligomers), the reaction proceeds to an exponential phase, with rapid formation of higher molecular mass fibrils. If there is addition of a seed (vertical arrow), the lag phase is reduced.

PMDs are usually attributed to proteins that are able to convert their native structure into β-sheet rich aggregates under certain circumstances. PMDs are characterized by the presence of a combination of mature fibrils, protofibrils and oligomers, which accumulate in the intra- or the extracellular environment of affected tissues [43–45] (Figure 3A). This wide group of PMDs includes several neurodegenerative diseases, such as AD (Alzheimer's disease), Parkinson's diseases, prion diseases [also known as TSEs (transmissible spongiform encephalopathies] and ALS (amyotrophic lateral sclerosis), among others. Although there is still a lot to learn about the mechanisms involved in these diseases, the growing number of maladies related to the protein misfolding phenomenon has given rise to an increasing knowledge on the subject.

Protein misfolding can occur at many different stages between the synthesis of a new protein and its degradation. Protein misfolding occurs due to gene mutations, which lead to an inability of the produced protein to correctly fold, malfunction of the protein trafficking machinery and abnormal protein interaction with different molecular partners in the cellular *milieu* [10,44,46,47]. The aggregates can be formed intracellularly, as is the case with α-synuclein (Parkinson's disease), SOD (superoxide dismutase) (in ALS) and huntingtin (Huntington's disease) [43] or extracellularly, as is the case in prion diseases [48,49], Type 2 diabetes [due to the aggregation of the IAPP (islet amyloid polypeptide)] [50,51] and AD [43].

The pathogenic role of these protein aggregates has long been discussed. Although large aggregates are the morphological markers of these diseases, they appear to be less toxic than oligomers, which display strong toxicity when incubated with cultured cells or inoculated into animals [52–54]. Oligomers usually progress in size and shape to form protofibrils and fibrils [55] (Figures 3A and 3B). Protein misfolding, accompanied by an increase in β-sheets, is generally associated with amyloid fibril formation, as they have a common cross-β scaffold [55,56]. A seeding-nucleation process follows in which the initial steps are slow and thermodynamically unfavourable [44], represented by a lag phase (Figure 3B). Oligomeric or protofibrilar species are formed during the lag phase; those protofibrils are called seeds. If a pre-formed seed is added to a new nucleation reaction, the lag phase is significantly reduced, reflecting increased aggregation kinetics (Figure 3B) [44].

The prion concept was developed by Stanley Prusiner in the 1980s to explain the existence of a group of transmissible diseases with a completely new transmission agent [57]. This phenomenon was detected many years before in sheep [58,59], but the prion concept could only be proven when Prusiner demonstrated that a protein in a misfolded conformation could act as an infectious agent and promote the conformational change of a protein with the native conformation, leading to disease propagation without the need of any other agent, particularly one containing a coding nucleic acid [57,60]. In the case of sporadic prion diseases, the generation of the misfolded version of the protein is still controversial, but it could include the participation of a co-factor [10,61,62].

Recently, a new view on this subject has become more popular with the discovery that several other proteins, such as Aβ (amyloid β-peptide), tau and α-synuclein, can behave as prions, demonstrated through their transmissibility using either mammalian cell cultures or animals [63–66]. *In vivo* evidence has been found in patients with Parkinson's disease submitted to transplant of fetal mesencephalic dopaminergic neurons, in which the formation of α-synuclein-positive Lewy bodies was detected in grafted cells [67]. These findings lead to a wider interpretation of the prion concept and may lead to the inclusion of other amyloid diseases in the group of prion diseases. Based on this concept, the next topic will focus on the misfolding and aggregation of p53 and its relation to protein malfunction and cancer.

### p53 misfolding and aggregation: amyloids and the prion-like behaviour

Formation of high-molecular-mass species of p53 was described in the early 1990s [68]. By this time, p53 was already known to be related to cell-malignant transformations, although it was believed that the aggregated form of p53 was a quaternary structure produced to prevent its rapid degradation. It was observed that p53 forms high-molecular mass aggregates through self-aggregation or through interaction with various cellular and viral proteins [69,70].

**Figure 4:**
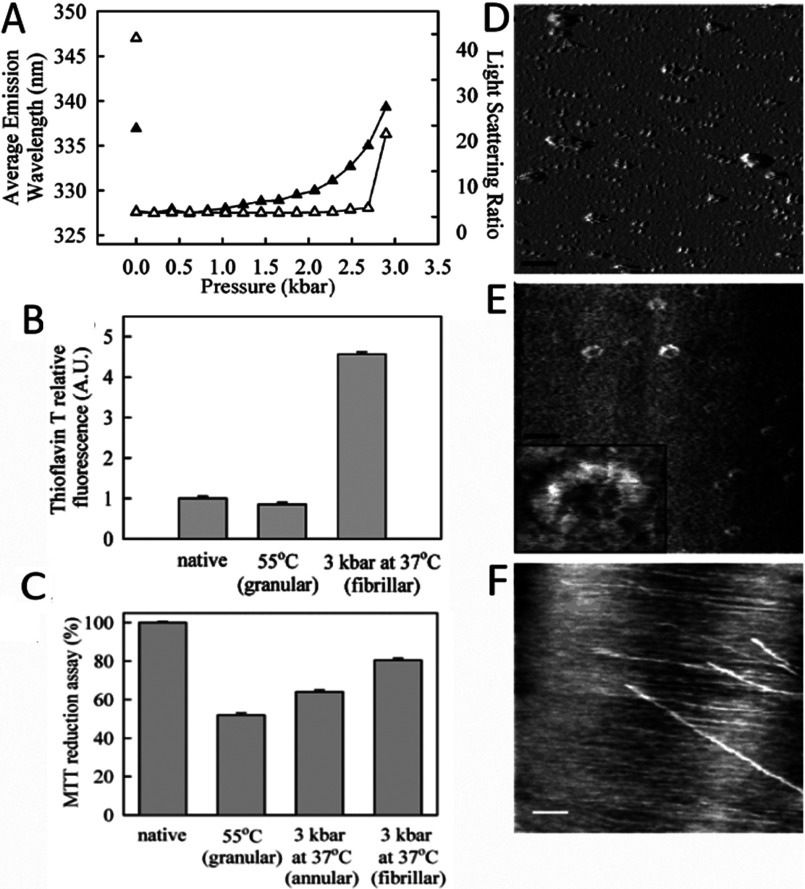
Aggregation of p53C induced by high pressure and high temperature (**A**) FI (intrinsic fluorescence) emission area (filled triangles) or LS (light scattering) values (empty triangles) of p53C at 5 μM during pressure treatment up to 3 kbar at 37°C. Isolated symbols at the left represent LS or FI values after return to atmospheric pressure. (**B**) Thioflavin T binding to the different p53C aggregated species obtained either by high temperature (55°C) or high pressure treatment. (**C**) MTT reduction assay reveals that p53C aggregates are toxic to cells (RAW macrophages) in culture. (**D–F**) AFM images of heat-induced p53C aggregates (**D**) pressure-induced annular p53C aggregates (**E**) and fibrilar aggregates of p53C 1 month after return to atmospheric pressure (**F**). Reprinted with permission from (Ishimaru D, Andrade LR, Teixeira LS, Quesado PA, Maiolino LM, Lopez PM, Cordeiro Y, Costa LT, Heckl WM, Weissmüller G, Foguel D, Silva JL (2003) Fibrillar aggregates of the tumor suppressor p53 core domain Biochemistry 42(30) 9022–9027). Copyright (2003) American Chemical Society.

The instability of p53 has been the subject of several biophysical studies, with denaturation being reached by chemical, thermal and pressurization means [16,71–74]. Our group demonstrated the formation of different types of aggregates (fibrillar, annular or granular) after physical-induction of unfolding of the p53 central core domain (p53C) [16,73]. Mild denaturation of p53C by pressure generated fibrillar aggregates (Figure 4), which were characterized by AFM (atomic force microscopy) and binding to thioflavin T was observed [16] (Figures 4A, 4B and 4F). Pressure treatment also produced annular aggregates that were observed immediately after decompression (Figure 4E); such aggregates were toxic to cells in culture, as shown by an MTT [3-(4,5-dimethylthiazol-2-yl)-2,5-diphenyl-2*H*-tetrazolium bromide] reduction assay (Figure 4C). Heat denaturation generated granular-shaped aggregates (Figure 4D) that were more cytotoxic than the pressure-induced aggregates [16] (Figure 4C).

**Figure 5:**
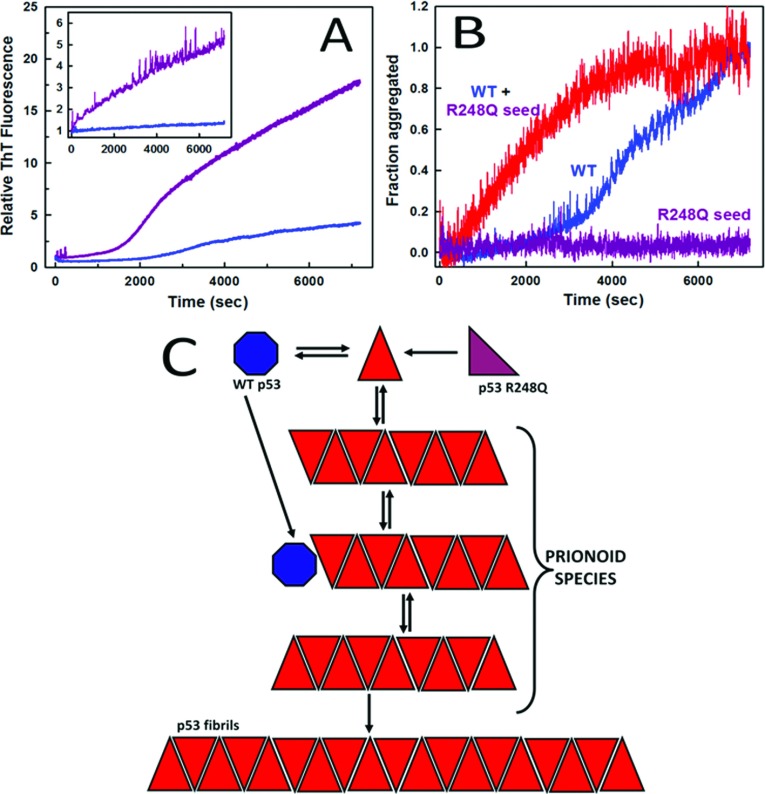
Dominant-negative phenomenon and gain-of-function prion-like effect (**A**) Aggregation kinetics of WT (blue) or R248Q p53C (purple) at 37°C at pH 7.2 or 5.0 (inset). (**B**) Aggregation of WT p53 at 10 μM without a seed (blue) or seeded with aggregated R248Q (red); R248Q was seeded alone at 2 μM as a control (purple). Aggregation of WT p53 or R248Q was monitored by thioflavin T binding at 37°C for 30 min. (**C**), Scheme showing conversion of native WT p53 (blue) or R248Q (purple) into a misfolded species (red triangle) that will further aggregate. The prionoid species are oligomers and protofibrils that bind anti-oligomer antibody. Adapted from Journal of Biological Chemistry: Ano Bom AP, Rangel LP, Costa DC, de Oliveira GA, Sanches D, Braga CA, Gava LM, Ramos CH, Cepeda AO, Stumbo AC, De Moura Gallo CV, Cordeiro Y, Silva JL. (2012) Mutant p53 aggregates into prion-like amyloid oligomers and fibrils: implications for cancer 287 (33): 28152–28162.

**Figure 6:**
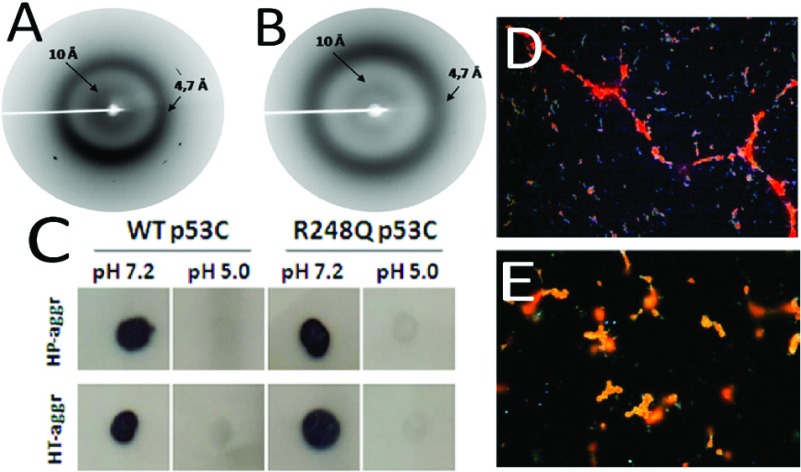
WT and R248Q p53 aggregate into amyloid fibrils X-ray diffraction spectra of high-pressure induced aggregates of WT (**A**) and R248Q (**B**) p53C at pH 7.2. (**C**) dot-blot assay with A11 antibody for pressure- and temperature-induced aggregates of WT and mutant p53C. Congo red birefringence visualized under polarized light of temperature-induced aggregates of WT (**D**) and R248Q (**E**) p53C. Magnification: ×400. Adapted from Journal of Biological Chemistry: Ano Bom AP, Rangel LP, Costa DC, de Oliveira GA, Sanches D, Braga CA, Gava LM, Ramos CH, Cepeda AO, Stumbo AC, De Moura Gallo CV, Cordeiro Y, Silva JL. (2012) Mutant p53 aggregates into prion-like amyloid oligomers and fibrils: implications for cancer 287 (33): 28152–28162.

The central core domain of p53 is the main target for mutations; more than 90% of hot-spot mutations in p53 occur in p53C [7,32]. We have shown that hot-spot mutants have a greater tendency to aggregate than WT p53 (Figure 5) [20,73]. Using different techniques, such as X-ray diffraction, electron microscopy, FTIR (Fourier-transform infrared) spectroscopy, DLS (dynamic light scattering), cell viability assay and anti-amyloid immunoassay, we demonstrated the amyloid nature of the aggregates (Figure 6) [20].

Apart from the central core domain, the transactivation and tetramerization domains have also been shown to form amyloid aggregates [17,18,75]. It was shown that unlike the WT p53 tetramerization domain, the G334V mutant forms amyloid fibrils by a two-step process under physiological temperature and pH conditions [75]. Additionally, this mutant was capable of forming heterotetramers with WT p53. Aggregate formation and protein conformational changes were evaluated by CD, binding of thioflavin T, AFM and Congo red birefringence [75]. The transactivation domain (residues 1–63), when exposed to low pH, formed aggregates enriched in β-sheets, presenting an amyloid pattern as shown by thioflavin T-binding, AFM and X-ray diffraction [18]. Taken together, these findings suggested that the whole protein could aggregate into amyloid assemblies, which was later demonstrated by several groups [19,20,76,77].

**Figure 7:**
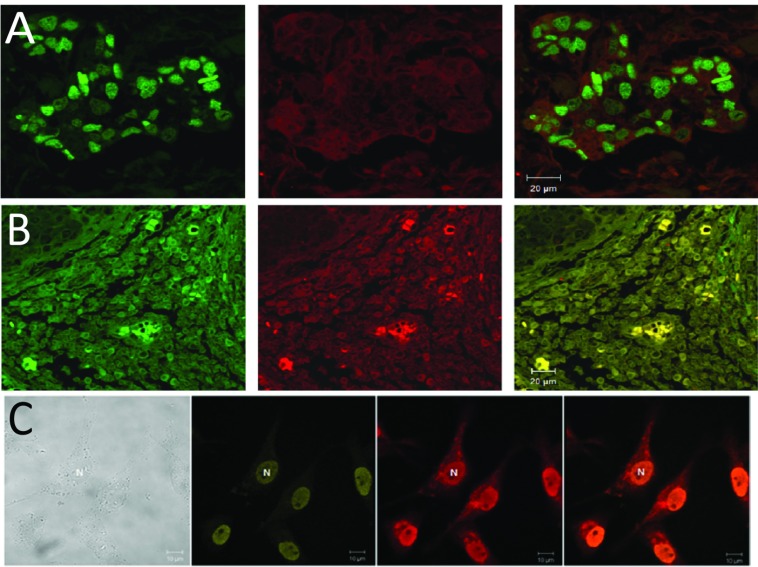
Tumour samples bear oligomeric species of WT and mutant p53 (**A**) WT p53 (green) and A11 anti-oligomer antibody (red). (**B**) Colocalization of mutant R175H p53 (green) with A11 anti-oligomer antibody (red). (**C**) MDA-MB231 breast cancer cells labelled with antibody against p53 (2nd column), with A11 Ab (3rd column) and the merge of second and third columns (fourth column) reveals nuclear aggregates of mutant p53. Magnification: ×400. Reprinted from The International Journal of Biochemistry & Cell Biology, 43(1), Levy CB, Stumbo AC, Ano Bom AP, Portari EA, Cordeiro Y, Silva JL, De Moura-Gallo CV, Co localization of mutant p53 and amyloid-like protein aggregates in breast tumors, 60–64, Copyright (2011), with permission from Elsevier. Adapted from Journal of Biological Chemistry: Ano Bom AP, Rangel LP, Costa DC, de Oliveira GA, Sanches D, Braga CA, Gava LM, Ramos CH, Cepeda AO, Stumbo AC, De Moura Gallo CV, Cordeiro Y, Silva JL. (2012) Mutant p53 aggregates into prion-like amyloid oligomers and fibrils: implications for cancer 287 (33): 28152–28162.

We detected p53 aggregates in archived samples of breast cancer tissues expressing mutant R248Q and other p53 hot-spot mutants using co-localization assays with A11 and anti-p53 DO1 antibodies [20,76] (Figure 7). In general, there was a strong correlation between tumour aggressivity and p53 aggregation. In the case of the breast tissue biopsy bearing R248Q, the patient had an invasive ductal carcinoma of Elston grade 3 [20].

We also found co-localization of full-length p53 and aggregates in tumour cell lines [20]. In the case of MDA-MB 231 cells (that express the R280K p53 mutant), there was massive aggregation in the cell nucleus [20] (Figure 7). When these aggregates were extracted from the cells, they eluted in the void volume of a gel-filtration column.

APRs (aggregation-prone regions) are found in most proteins. These small APRs are normally sheltered from aggregation due to intra-protein interactions or because they are hidden in the hydrophobic protein core. However, when the protein structure is disturbed by mutations or environmental changes, these sequences can be exposed, thus favouring protein aggregation [78]. One of these sequences has been detected within the p53 hydrophobic core of the DBD by Xu et al. [19]. This sequence is exposed when several mutations occur and leads to aggregation of p53. This aggregation inactivates not only the product of the WT allele of the *TP53* gene but also other proteins belonging to the p53 family, p63 and p73, which have very similar aggregating-prone sequences that facilitate co-aggregation. Perinuclear aggregates formed after transfection with mutant p53 were detected through immunostaining and confocal microscopy. These results were confirmed in different types of tumours, such as human colon adrenocarcinoma, mouse lung metastases from osteosarcoma and mouse kidney lymphoma [19]. This co-aggregation abrogates the p53 paralogous function, a consequence of the characteristic gain-of-function role of these mutants.

The formation and kinetics of WT and mutant p53 amyloid oligomers and fibrils has been widely discussed [20,79–82]. p53 aggregates have been detected in different types of cancer biopsies, such as breast cancer [20,76] and basal cell carcinoma [80]. Additionally, cholesterol secosterol aldehydes, which are lipid-derived aldehydes frequently detected in chronic inflammation, appear to play a role in the formation of p53 amyloid aggregates [77]. Moreover, p53 function appears to be lost during incubation with these compounds, thus showing a possible interplay between chronic inflammation and cancer development, which is often reported in epidemiological studies [77].

**Figure 8:**
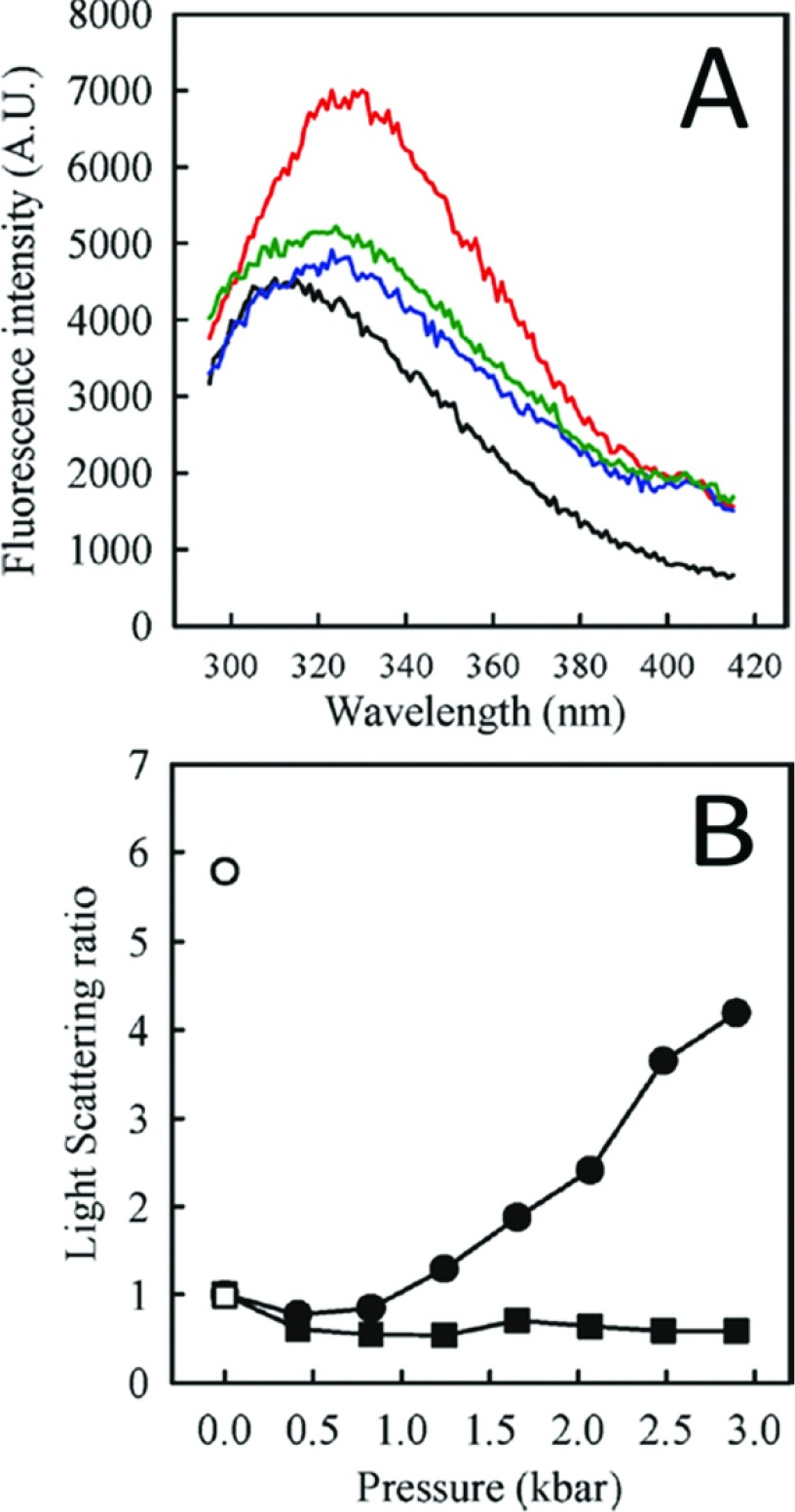
Cognate DNA rescues native conformation of p53C (**A**), Fluorescence emission spectrum of p53C at 1 atm (black line); after a compression and decompression cycle (up to 3 kbar) in the absence of DNA (red line); after DNA addition at 1 atm (blue line); after a second compression and decompression cycle in the presence of DNA (green line). (**B**) Light scattering ratio for the experiment shown in (**A**) in the absence of DNA (circles) and after compression/decompression after addition of consensus DNA (squares). Open symbols correspond to LS values after return to atmospheric pressure. Reprinted with permission from (Ishimaru D, Ano Bom AP, Lima LM, Quesado PA, Oyama MF, de Moura Gallo CV, Cordeiro Y, Silva JL (2009) Cognate DNA stabilizes the tumor suppressor p53 and prevents misfolding and aggregation. Biochemistry 48(26) 6126–6135) Copyright (2009) American Chemical Society.

The DNA-binding properties of p53 are frequently lost upon p53 aggregation; however, the small cognate double-stranded DNA has been shown to stabilize both the p53C domain and full-length p53 and to have the potential to rescue aggregated and misfolded species [83] (Figure 8). Therefore, such DNA sequences could be exploited as a new approach to cancer therapy in the near future. The use of small aptameric nucleic acids and other polyanions has been proposed to modulate aggregation of the prion protein [47,62,84]. On the other hand, p53 has been previously described to form long fibrillar segments complexed with DNA [85].

The presence of intermediate states that participate in the p53 aggregation route has been highlighted in different studies [8,72,86]. Mutants are unstable and appear to unfold and aggregate faster than the WT form of p53 [20]. An intermediate conformation of p53C during equilibrium and kinetic folding/unfolding transitions induced by GdmCl (guanidinium chloride) has been demonstrated [74]. Moreover, experiments combining high hydrostatic pressure and sub-zero temperatures [73] were able to evidence an intermediate state with a fold similar to the hot-spot mutant, R248Q, as demonstrated by NMR. These data indicate a finely energetically regulated mechanism for the formation of p53 aggregates, which, at first glance, may appear random. New investigations in this area might provide a better characterization of this pathway. The regulation of this process is definitely promising for the modulation of p53 aggregation in cancer cells.

The dominant-negative hypothesis for WT p53 inactivation in cells carrying a mutation in one of the alleles of the *TP53* gene proposes that the presence of even a single mutant protein in the formation of the p53 tetramer would lead to loss-of-function of p53 [87,88]. Our group has previously proposed an alternative hypothesis for the dominant-negative effect in which WT p53 at lower concentrations would be incorporated into aggregates containing the mutant p53 (Figures 5B and 5C) [10,16]. In our hypothesis, the presence of a misfolded conformation of mutant p53 would sequester the properly folded form, abrogating its function, in a prion-like mechanism. We found that R248Q mutant oligomers and fibrils could seed aggregation of WT p53 [20] (Figure 5B). The lag phase of WT p53 aggregation was suppressed demonstrating the high seeding potential of the aggregated mutant protein.

As represented in the diagram (Figure 5C), mutant p53 would convert the WT form into a faster aggregating species [20]. Hetero-oligomerization would more likely occur in smaller aggregates, and the formation of fibrils would make aggregation an irreversible phenomenon. These findings are also in line with the results that the anti-oligomer antibody bound more targets in tumour tissues containing the p53 mutations [20,76].

A misfolded pathogenic species would act as a molecular chaperone inducing the correctly folded form of p53 to adopt a misfolded conformation, a seed that would then increase aggregation exponentially. The higher susceptibility to aggregation of mutant p53 would amplify this process [20]. We propose that the aggregated form of mutant p53 may act as a sink, sequestrating the native protein into the inactive conformation through a mechanism described for a typical prionoid [63,64]. This reinforces the proposal that cancer should be considered, at least in part, as a prion disease [10,20]. Finally, the possibility of modulation of this phenomenon [82,83] opens up new possible targets for cancer chemotherapy.

### The participation of other proteins on p53 aggregation and formation of intracellular aggregates and aggresomes

As mentioned before, misfolded forms of p53 can occur either due to mutations or to situations in which p53 is inactivated. As described here, the co-aggregation of p53 and its paralogues, p63 and p73, were demonstrated intracellularly [19]. The erroneous interaction of p53 with the chaperone Hsp70 (heat-shock protein 70) and their interaction with Mdm2 promoted the formation of misfolded aggregates of p53, giving rise to structures named ‘pseudo-aggregates’ with β-amyloid characteristics [89]. It was proposed that these interactions may stabilize p53 mutants in cells. Hsp90, on the other hand, appears to interact with p53 and convert it to a molten globule state [90], a conformation that is also observed when p53 is incubated at low pH [91]. We also found p53 in acidic compartments of human breast cells in culture [91], which combined with the results that p53 at low pH has less tendency to aggregate [20] indicate that the cell has several strategies to prevent p53 aggregation.

In addition to self-aggregation and combined mutant-WT aggregation, p53 aggregation has also been described where other proteins function as a platform. This is the case for the above mentioned p53–Hsp70–Mdm2 interaction [89] and also for the acetyltransferase p300, a protein that contains a highly disordered region that displays similarities to prion-like domains. p300 has been shown to provide an interaction interface for various misfolded proteins, including p53, promoting their aggregation. Moreover, the down-regulation of this protein impairs proteasome activity and enhances toxicity caused by the stress of induced protein misfolding, indicating a physiological role for p300 in protein uptake for proteasome degradation [92]. The use of proteasome inhibitors induced the formation of nucleolar aggresomes, containing p53, pRb (retinoblastoma protein), poly(A)^+^ RNA (polyadenylated RNA), conjugated ubiquitin and several cell cycle-regulating cyclins, among other molecules [93]. p53 was also found in conjunction with a set of proteins in aggregates formed in the necrotic core of multicellular tumour spheroids [94].

### Concluding remarks

We conclude that aggregation of p53 into a mixture of oligomers and fibrils sequesters the native protein into an inactive conformation that is typical of a prionoid. Our findings that amyloid aggregates are present in biopsies of breast cancer tissues, especially in the aggressive tumours, show the relevance of this prion behaviour in cancer pathogenesis [20,76]. In fact, the possible role of protein-only inheritance and prions in cancer has been recently discussed by Antony et al. (2012) [95]. In their review, the authors highlighted the genetic studies with yeast showing a large number of proteins as prions that confer dominant phenotypes with cytoplasmic inheritance. They point out that many of these proteins have mammalian functional homologues. More recently, another tumour suppressor, retinoblastoma RB AB domain, was shown to have similar aggregation properties to p53 tumour suppressor [96], which is particularly relevant since both cell regulators are inactivated in most cancers.

The prion-like behaviour of oncogenic p53 mutants, as represented in Figure 5(C), provides an explanation for the dominant-negative effect and the gain of function, including the high metastatic potential of cancers bearing p53 mutations. The inhibition of aggregation of p53 into oligomeric and fibrillar amyloids by nucleic acid aptamers and small molecules appears to be a good target for therapeutic intervention in tumour diseases.
